# Computerized cognitive training improves cognitive function in primary breast cancer survivors

**DOI:** 10.1038/s41523-024-00694-8

**Published:** 2024-09-30

**Authors:** Karl R. Kleinknecht, Mira Bierend, Lisa-Maria Keim, Frederik Bartels, Amit Lampit, Carsten Finke

**Affiliations:** 1 Department of Neurology, Charité - Universitätsmedizin Berlin, Berlin, Germany; 2 Berlin School of Mind and Brain, Humboldt-Universität zu Berlin, Berlin, Germany; 3 Department of Psychiatry, The University of Melbourne, Melbourne, VIC Australia

**Keywords:** Breast cancer, Chemotherapy, Pain

## Abstract

Cancer-related cognitive impairment has a significant impact on the quality of life and perceived cognitive ability of breast cancer patients and frequently affects attention, working memory, and executive function. Several interventional approaches to treat these deficits have been studied, including web-based cognitive training, but methods and timing in relation to cancer treatment are heterogeneous. Only few interventions start early after primary breast cancer treatment, a time when many patients report the greatest impairments in quality of life and cognition. In this randomized controlled pilot study, 31 breast cancer survivors with subjective cognitive deficits and a mean post-treatment duration of 6.6 months (SD = 9.3) were assigned to either 14 weeks of a web-based cognitive training program (training group, *n* = 16) or a control group (*n* = 15). All patients underwent detailed neuropsychological assessment, evaluation of patient-reported outcomes (PROMs), and neurological examination before (baseline, T1) and after (follow-up, T2) the intervention. Longitudinal (T1 vs. T2) and cross-sectional (T2) cognitive performance was assessed for both groups. Overall cognitive impairment significantly improved in the training group following training (56% vs 25%; *p* = 0.03, Phi = 0.51), but not in the control group (73% vs. 73%; *p* = 1) in the longitudinal analysis (T1 vs. T2). Specifically, the training group showed statistically significant improvement of executive functions (*p* = 0.004, Phi = 0.32). No effects of training on subjective cognitive deficits or PROMs were observed. Comparing cross-sectional cognitive performance at follow-up (T2), the training group showed a significantly lower rate of cognitive impairment overall (*p* = 0.007, Phi = 0.48) and a better cognitive performance for executive function (*p* = 0.04, Phi = 0.32) compared to the control group. In this prospective pilot study, web-based cognitive training was efficacious in improving overall cognitive performance and executive function. Importantly, this study investigated a web-based cognitive training for the immediate post-treatment phase, when up to 75% of breast cancer patients experience cognitive decline. These results indicate that cognitive training may improve neuropsychological outcomes for patients with breast cancer.

## Introduction

Cancer-related cognitive impairment (CRCI) is a common symptom in breast cancer (BC) survivors and is reported by up to 75% of patients^1–4^. CRCI frequently affects the cognitive domains of memory, attention, and executive function^3,5,6^ with mild to moderate severity^2^ and has a substantial negative impact on the quality of life of cancer patients^6–8^. Up to 40% of BC patients experience CRCI even before the start of (adjuvant) therapy^2,4,9^, and up to 60% are suffering from cognitive impairment after treatment^2^. Although chemotherapy is likely to contribute to CRCI^2,6,10–12^, numerous other factors have been hypothesized. These include side effects of other treatments^13–16^ and tumor-related factors such as neuronal autoantibodies^17^. Indeed, 25% of BC patients harbor neuronal antibodies including antibodies targeting N-Methyl-D-Aspartate (NMDA) receptors^18^, and recent studies have shown an association of these neuronal antibodies with cognitive deficits in cancer patients^19–21^.

Only sparse evidence exists regarding treatments of CRCI^2,22,23^. While the effect of pharmacological interventions remains limited^2,22^, behavioral interventions show promising effects^2,22^. Studies investigating cognitive training in general and in cancer patients found that computerized cognitive training can improve cognitive function for the domains of processing speed, executive function, working memory, cognitive flexibility, language, and immediate and delayed memory^24–26^. Selected studies also reported an improvement following cognitive interventions for subjective cognitive function and quality of life^27^, however, most studies failed to find such an effect^28–30^.

Studies in patients with breast cancer using fully or partly computerized cognitive training likewise indicate improvements mainly for the domains of memory, processing speed, verbal learning, and working memory^27,31,32^, some of them additionally indicating transfer effects from trained cognitive domains to real-life tasks^27,31^. Heterogeneity for results in existing studies in breast cancer patients is mainly due to the question of effectiveness of computerized cognitive training on perceived subjective cognitive impairment—some indicate significant improvement^29,33^, others do not^32^. Additionally, methods, duration, and intensity of cognitive training as well as neuropsychological assessment vary considerably between these studies. The duration of the training period described for existing studies varies between 4 to 8 weeks with a total training duration of 2 up to 48 hours; cognitive interventions in breast cancer patients have so far mainly been studied with an average timing of 3 to 6 years after completion of (chemo)therapy and with a mandatory minimum delay of 6 to 18 months after the end of primary breast cancer therapy^27,29,31–33^, although several studies indicate that reduction in quality of life and perceived cognitive abilities is most severe during treatment and in the early period after cancer treatment and improves over years^2,4,34,35^, thus interventions should ideally target these early disease and therapy stages and should start early after treatment initiation.

Here, we examined the effect of a web-based cognitive training in the immediate post-treatment phase (within 0–30 months of completion of primary therapy) across 14 weeks with 3 training sessions/week in breast cancer patients on i) cognitive impairment in the most frequently affected cognitive domains in breast cancer patients (i.e., memory, attention, executive function) and ii) subjective cognitive impairment and patient-reported outcome measures including quality of life, depression, and fatigue.

## Results

### Baseline (T1)

Demographic data between the control and training group were similar at T1, especially for age (mean age ± SD: control group 54.4 ± 13.8 vs training group 53.7 ± 11.3 years, *p* = 0.83) and years of education (mean ± SD: control group 15.3 ± 2.3 vs training group 14.5 ± 2.1, *p* = 0.318) (Table 1). Similarly, tumor and treatment characteristics were comparable (Table 1): Around 90% of patients in both groups had tumors with lower UICC stages (UICC ≤ 1: control group 47% vs. training group 63%, *p* = 0.376; UICC 2a/2b: control group 40%, training group 31%, *p* = 0.611, Table 1) and the groups had similar rates of non-cancer disorders from an array of clinical areas (Supplementary Table 1); all patients had completed surgery and frequencies of other treatment forms (e.g., chemotherapy, radiotherapy, antibody therapy, hormone therapy) did not differ between the training and control group, except hormone therapy (hormone therapy: control group 100% vs. training group 75%, *p* = 0.038, Table 1), although the number of patients that received current hormone therapy at T1 was also similar for both groups (current hormone therapy at T1: control group 60% vs. training group 50%, *p* = 0.576, Table 1). For chemotherapy, 2 control group and 3 training group patients had not yet fully completed chemotherapy at T1 and one patient of each group was still receiving radiation therapy; for these, the neuropsychological assessment took place between the cycles and at least 10 days after administration. Groups were also comparable regarding baseline quality of life measures and oncological status (Functional Assessment of Chronic Illness Therapy—Fatigue (FACIT-Fatigue), Beck Depression Inventory—Fast Screen (BDI-FS), 12-Item Short Form Health Survey (SF-12), Eastern Cooperative Oncology Group scale of performance status (ECOG), Karnofsky performance status (Karnofsky), Table 2) as well as for the meantime since therapy (Table 1).

**Table 1:** Baseline demographic and clinical characteristics of the control and the training group

	Control group (*n* = 15)			Training group (*n* = 16)			
		*n*	%		*n*	%	*p*-value
Age mean (years)	54.4 (SD 13.8)			53.6 (SD 11.2)			0.830
Age range (years)	30–75			39–78			
	≤39	3	20		1	6	0.254
	40–49	1	7		5	31	0.083
	50–59	6	40		7	44	0.833
	>59	5	33		3	19	0.354
Years of education	15.3 (SD 2.3)			14.5 (SD 2.1)			0.318
	≤13	5	33		6	38	0.809
	>13	10	67		10	63	0.809
IQ (MWTA, mean)	118.7 (SD 12.6)			123.4 (SD 11.0)			0.299
UICC tumor stage at diagnosis							
	≤1	7	47		10	63	0.376
	2a/2b	6	40		5	31	0.611
	3a	2	13		1	6	0.505
Human epidermal growth factor receptor 2 positive		5	33		5	31	0.901
Time since therapy mean (months, *n* = 29)	5.07			8.07			0.715
Time since therapy (min–max)	0-24			0–30			
Surgery		15	100		16	100	–
Chemotherapy		14	93		12	75	0.165
Current chemotherapy at T1		2	13		3	19	0.682
Radiotherapy		12	80		12	75	0.739
Current radiotherapy at T1		1	7		1	6	0.962
Antibody therapy		6	40		5	31,3	0.611
Current antibody therapy at T1		3	20		2	13	0.570
Hormone therapy		15	100		12	75	0.038
Current hormone therapy at T1		9	60		8	50	0.576

**Table 2:** Neuropsychological performance data and quality of life measures

	Control group (*n* = 15)		Training group (*n* = 16)	
	T1 mean (SD)	T2 mean (SD)	T1 mean (SD)	T2 mean (SD)
SF12 score physical health	38.6 (9.5)	40.4 (11.0)	41.3 (7.8)	43.6 (10.5)
SF12 score mental health	46.1 (11.5)	44.4 (11.8)	46.4 (10.7)	49.9 (11.1)
Karnofsky	80.7 (12.2)	78.7 (8.3)	78.1 (8.3)	79.4 (10.0)
ECOG	0.7 (0.6)	0.9 (0.5)	0.8 (0.4)	0.7 (0.5)
Fatigue subscale score	29.9 (10.7)	33.1 (11.0)	34.1 (9.5)	35.3 (9.7)
BDI FS T-score	61.4 (7.7)	60.5 (9.2)	59.5 (7.2)	59.1 (6.6)
FACT-Cog PCI	37.9 (14.0)	45.4 (14.6)	42.6 (17.5)	50.8 (12.1)
FACT-Cog QOL	8.8 (4.6)	10.3 (4.1)	10.0 (3.0)	11.4 (4.2)
FACT-Cog OTH	14.4 (2.1)	14.2 (3.1)	15.1 (1.3)	15.1 (1.5)
FACT-Cog PCA	15.0 (5.1)	15.9 (4.4)	15.3 (5.1)	18.1 (4.5)
FACT-Cog cognitive function score	76.1 (21.3)	85.7 (23.5)	82.9 (24.2)	95.4 (19.4)
VLMT sum score	58.3 (7.5)	59.5 (7.7)	59.9 (8.6)	61.9 (8.2)
VLMT immediate memory	7.4 (1.9)	8.1 (2.5)	7.8 (2.2)	8.1 (2.4)
VLMT best learning	13.7 (1.6)	14.0 (1.6)	13.9 (1.7)	14.6 (0.9)
VLMT susceptibility to interference	11.9 (2.4)	12.6 (2.4)	12.9 (2.8)	13.3 (2.3)
VLMT delayed recall	13.0 (1.9)	13.0 (1.8)	12.8 (2.5)	13.4 (2.0)
VLMT recognition	13.9 (1.2)	13.7 (1.6)	14.2 (1.2)	14.0 (2.9)
ROCF immediate recall	24.7 (5.7)	30.2 (2.9)	22.7 (4.8)	28.8 (4.7)
ROCF delayed recall	24.0 (5.7)	29.4 (3.5)	22.6 (4.2)	28.4 (5.1)
Digit span forward	7.9 (1.6)	8.1 (1.6)	7.5 (2.4)	8.1 (2.0)
Digit span backward	7.5 (1.7)	6.9 (1.7)	6.6 (1.5)	7.1 (1.7)
LPS subtest 3	25.5 (4.5)	28.7 (4.6)	26.4 (4.7)	29.5 (4.9)
Tonic Alertness median ms	299.6 (46.8)	305.6 (47.6)	299.5 (52.0)	283.3 (42.2)
Phasic Alertness median ms	305.3 (43.5)	328.1 (60.8)	302.2 (60.5)	277.3 (40.6)
Divided attention, auditive task	687.5 (79.6)	680.6 (81.7)	653.3 (117.7)	607.9 (99.4)
Divided attention, visual task	839.7 (99.0)	832.9 (81.0)	804.4 (89.0)	789.4 (57.4)
Divided attention, errors	2.3 (2.6)	2.9 (5.0)	2.5 (3.8)	2.3 (3.8)
Divided attention, omission	1.8 (1.8)	1.5 (2.0)	1.7 (1.4)	1.1 (1.1)
Go Nogo reaction time	611.9 (72.8)	624.3 (77.9)	603.3 (77.7)	605.5 (50.2)
Go Nogo, errors	2.0 (3.4)	0.1 (0.3)	0.5 (1.5)	0.7 (2.2)
GO Nogo omissions	0.5 (0.9)	0.1 (0.3)	0.6 (1.3)	0.3 (0.9)
Stroop time	123.4 (35.0)	113.9 (27.6)	113.6 (21.8)	98.3 (17.1)
Stroop errors	0.2 (0.6)	0.2 (0.4)	0.6 (1.5)	0.1 (0.3)
Verbal fluency	26.9 (5.1)	30.2 (7.7)	32.8 (6.7)	34.8 (5.5)
MWTA derived IQ	118.7 (12.6)	113.3 (15.3)	123.4 (10.9)	121.3 (13.4)

**Fig. 1:**
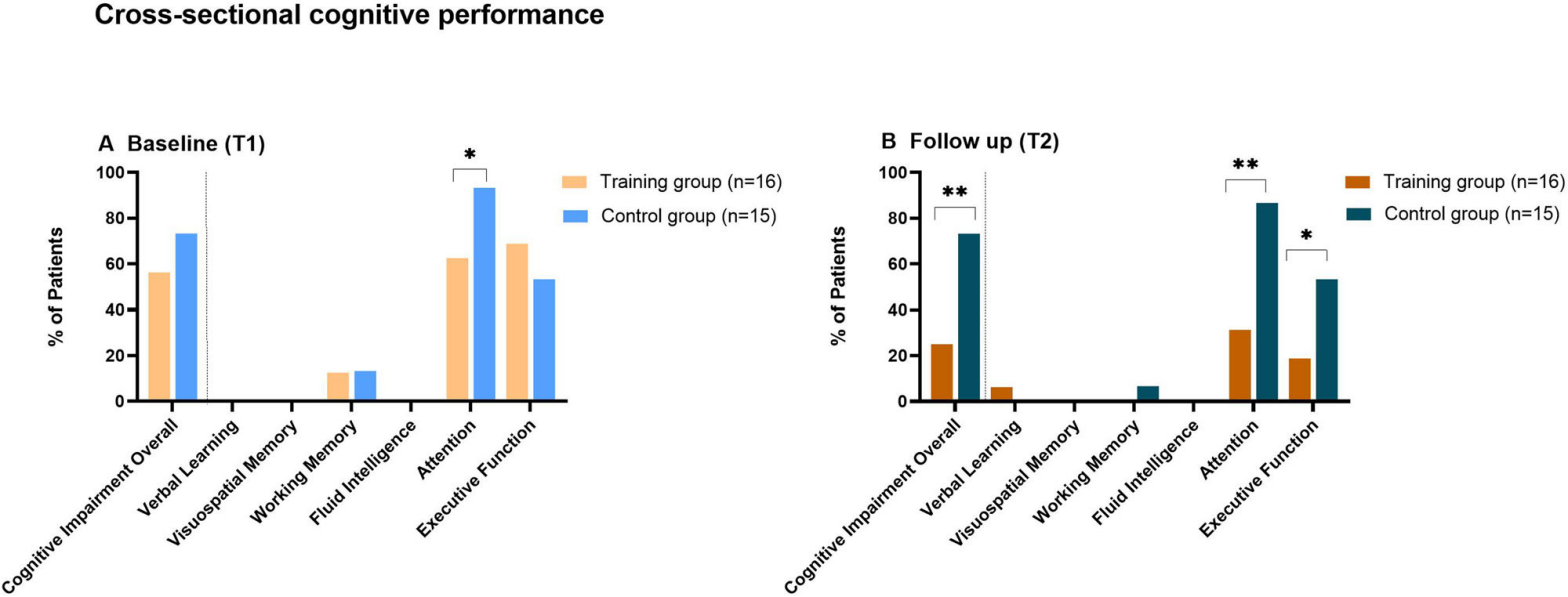
Cross-sectional cognitive performance. Group comparison between the training group (orange) and the control group (blue) for incidence of overall cognitive impairment and for impairment in cognitive domains. Tested cognitive domains were: verbal learning, visuospatial memory, working memory, fluid intelligence, attention, and executive function. **A** Baseline Timepoint 1 (T1) and (**B**) Follow-up Timepoint 2 (T2): the training group displayed fewer cognitive deficits for the domain attention (Chi-Square Test *p* = 0.002) and executive function (Chi-Square Test *p* = 0.04) and showed less Cognitive Impairment Overall (Chi-Square Test *p* = 0.007) compared to the control group at T2. T1 = Baseline/Timepoint 1; T2 = Follow-up/ Timepoint 2; SD = standard deviation; ** = *p* < 0.01, * = *p* < 0.05.

At baseline (T1), rates of overall cognitive impairment were not statistically different between the control and training group (control group 11/15 (73%), training group 9/16 (56%); *p* = 0.32). Patients showed cognitive deficits at baseline in the following domains: short-term memory (control group 2/15, 13.3%, vs. training group 2/16, 12.5%, *p* = 0.945), attention (control group 14/15, 93.3%, vs. training group 10/16, 62.5%, *p* = 0.04) and executive function (control group 8/15, 53.3%, vs. training group 11/16, 68.8%, *p* = 0.379; Fig. 1).

Three participants (3/34, 9%) did not complete the training. Training adherence of the remaining 31 participants was 100% across the full length of the web-based training program; all 40 sessions were completed.

### Longitudinal cognitive performance

**Fig. 2:**
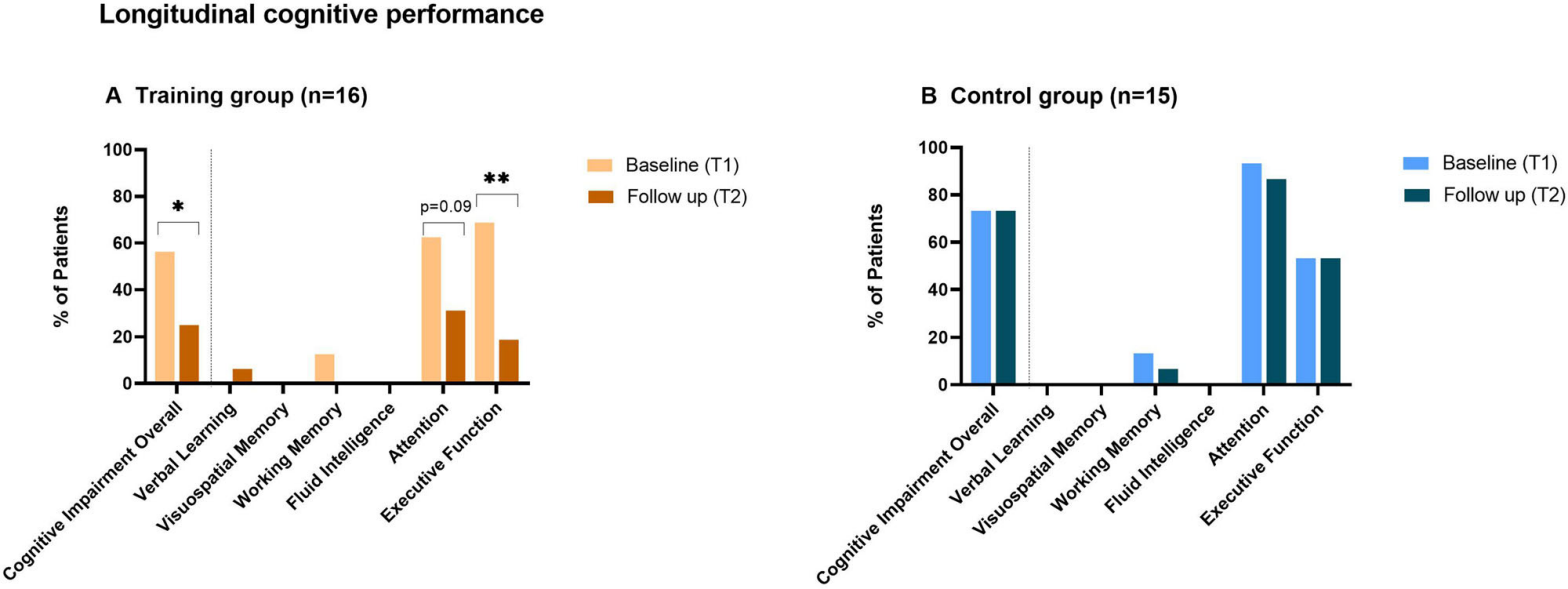
Longitudinal cognitive performance. Tested cognitive domains were: verbal learning, visuospatial memory, working memory, fluid intelligence, attention, and executive function. **A** Training Group: comparison of cognitive impairment overall and single cognitive domains between T1 (orange) and. T2 (red). Cognitive impairment declined from 56% to 25% of patients (McNemar Test *p* = 0.03). The training group improved for the cognitive domains executive function (McNemar Test *p* = 0.004) and attention (McNemar Test *p* = 0.09). **B** Control group: comparison of cognitive impairment overall and for single cognitive domains between T1 (light blue) and T2 (dark blue). Cognitive impairment overall and deficits for single cognitive domains remained equal from T1 to T2. T1 = Baseline/Timepoint 1; T2 = Follow-up/Timepoint 2; SD = standard deviation; ** = *p* < 0.01.

In the training group, the number of patients with overall cognitive impairment was reduced from 56.3% to 25.0% following 14 weeks of cognitive training (*p* = 0.03, Phi = 0.51, strong effect; Fig. 2). In contrast, in the control group, the number of patients with cognitive impairment did not change between T1 and T2 (73% vs. 73%; *p* = 1; Fig. 2). At the domain level, the training group showed significantly less deficits of executive function following the intervention (68.8% vs 18.8%, *p* = 0.004, Phi = 0.32, intermediate effect). Additionally, the training group also showed differences in the attention domain which were not significant (62.5% vs. 31.3%, *p* = 0.09). The level of executive function (53.3% vs 53.3%, *p* = 1) and attention (93.3% vs. 86.7%, *p* = 1) impairments remained unchanged in the control group (Fig. 2). No significant changes between T1 and T2 were observed for memory in either group.

### Cross-sectional cognitive performance (follow up/T2)

At follow-up (T2), the training group had a significantly lower rate of overall cognitive impairment compared to the control group (*p* = 0.007, Phi = −0.48, intermediate effect). At the domain level, patients in the training group had fewer deficits in the attention domain (*p* = 0.002, Phi = −0.56, strong effect) and the executive function domain (*p* = 0.04, Phi = 0.32, intermediate effect) compared to the control group (Fig. 1).

### Patient-reported outcomes

The training and control group did not differ significantly in measures of quality of life at T2, including SF-12, FACIT, BDI, ECOG, Karnofsky and FACT-Cogsubscale QOL.

**Fig. 3:**
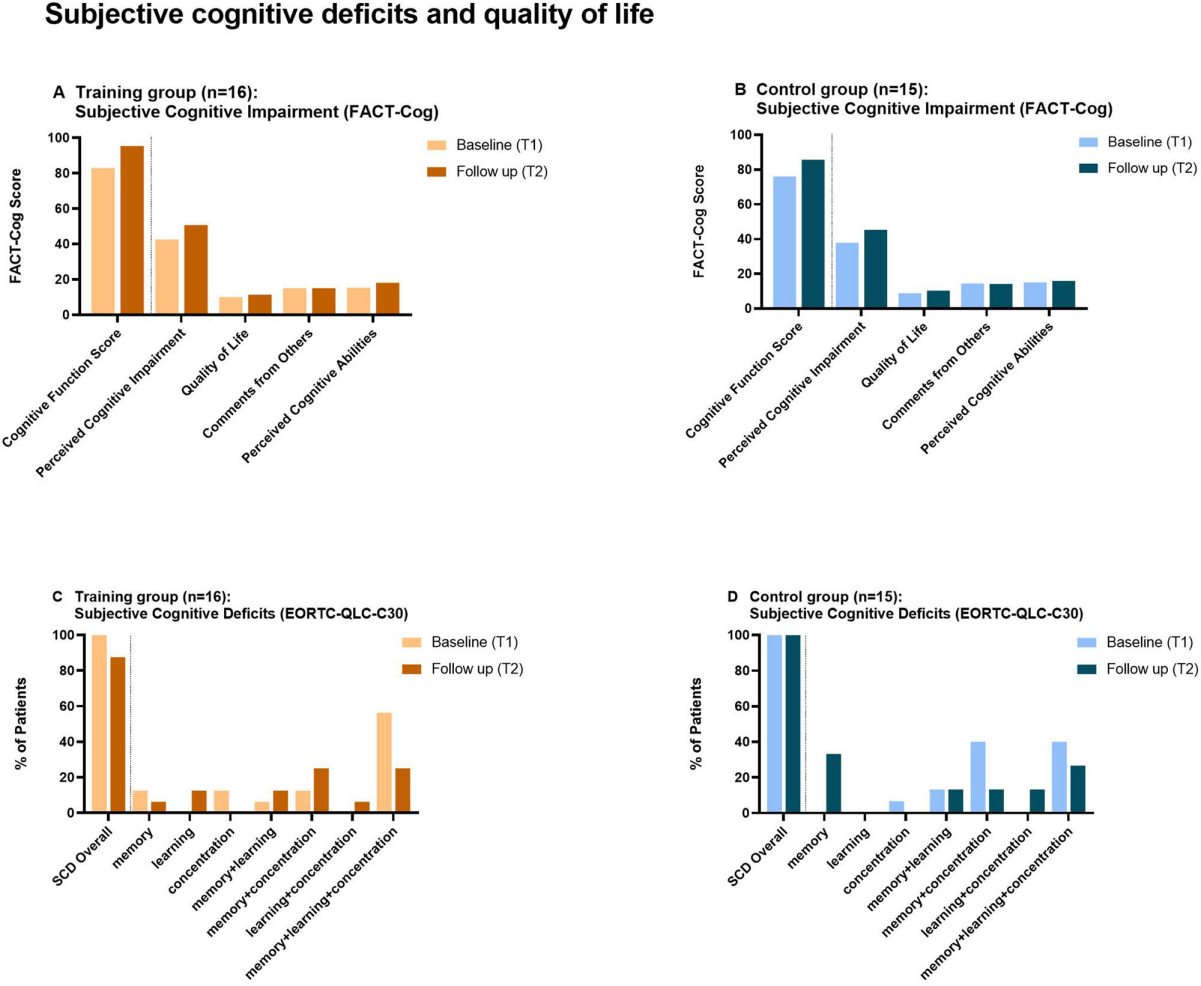
Subjective cognitive deficits and quality of life. **A** Comparison of FACT-Cog scores T1 versus T2 for the training group. **B** Comparison of FACT-Cog scores T1 versus T2 for the control group. Scores show small improvement from T1 to T2 for both groups. **C** Comparison of subjective cognitive deficits between T1 versus T2 for the training group. At T1 all participants complained about subjective cognitive deficits, at T2 the participants of the training group reported less subjective cognitive deficits [14/16 (87.5%, *p* = 0.157)]. Most prevalent at T1 was a combination of deficits for the domains memory, attention and learning (56.3%), at T2 only 25% of the training group described deficits in all three domains. **D** Comparison of subjective cognitive deficits between T1 versus T2 for the control group. At T1 and T2 all participants complained about subjective cognitive deficits. FACT-Cog = Functional Assessment of Cancer Therapy-Cognitive Function EORTC-QLC-C30 = European Organization for Research and Treatment of Cancer QLQ-C30; SCD = Subjective Cognitive Deficits; T1 = Baseline/ Timepoint 1; T2 = Follow-up/Timepoint 2.

Subjective cognitive impairment, measured with the FACT-Cog questionnaire, showed no significant improvement over time and no significant group differences (Table 2, Fig. 3). Comparing T1 vs. T2 within the training group, training had no effect on any of the FACT-Cog subscales or the FACT-Cog cognitive function score (Table 2). Additionally, subjective cognitive deficits (SCD) were assessed with a short semi-structured interview focused on general cognitive impairment in daily life, similar to items from the European Organisation for Research and Treatment of Core Quality of life Questionnaire (EORTC-QLC-C30, Item Number 20 and Item Number 25). SCD were reported by all patients in both groups at baseline (control group 15/15 (100%) vs. training group 16/16 (100%)) (T1). Most prevalent was a combination of SCD for memory + attention + learning (control group 6/15 (40%) vs. training 9/16 (56%), *p* = 0.366). At follow-up (T2), all 15/15 patients of the control group continued to report subjective cognitive deficits; in the training group, the number with SCD had declined to 14/16 (87.5%, *p* = 0.157, Fig. 3).

Participant satisfaction with the training, assessed using a short 4-item questionnaire adapted from Schmiedek et al.^36^, was high: 13/16 of the training group rated the training “fun”, 14/16 would recommend it to others and 15/16 would undergo the training again (Supplementary Table 2).

## Discussion

This prospective pilot study investigated the efficacy of a web-based cognitive training program on cancer-related cognitive impairment in breast cancer patients in the immediate post-treatment phase (mean 7 months). We observed a significant improvement for overall cognitive function and for executive functions at follow-up in the training group, but not in the control group. Our results show that web-based cognitive training may be efficient to ameliorate cancer-related cognitive impairment during the immediate post-treatment phase after primary breast cancer therapy.

CRCI affects up to 75% of breast cancer patients and negatively impacts numerous aspects of daily living, i.e., working performance and quality of life^1,2,4,6,37^. In line with previous studies^1,4^, we observed cognitive impairment for working memory, attention and executive function in our study population, while the verbal domain was not affected. In our study, the training group showed significant improvement following cognitive training in both longitudinal and cross-sectional analyses: Analyzing longitudinal change from T1 to T2 after the 14-week cognitive training, the training group performed significantly better than the control group in the executive function cognitive domain and showed significant improvement for overall cognitive function. Comparisons between the training and the control group following the cognitive training at T2 in cross-sectional analyses showed significantly better performance of the training group in the domains executive function and attention, and revealed significantly less overall cognitive impairment.

Previous studies that utilized behavioral as well as computerized cognitive training report conflicting evidence. Most studies observed improvements for attention, but also for executive function, working memory, memory, processing speed and visuospatial skills^2,27,31^. Kesler et al. observed better executive function skills like cognitive flexibility, verbal fluency and processing speed, using a web-based cognitive training for 12 weeks^31^. Damholdt et al. reported significant improvements for verbal learning and working memory after a 6 week computerized cognitive training^32^. In contrast, Mihuta et al. found no significant changes of memory or attention after a 4 weeks web-based and behavioral training^29^. A recent meta-analysis of subjective and objective effects of computerized cognitive training (CCT) in 9 RCTs involving 666 patients with breast cancer unexpectedly reported that cognitive training did not improve attention, executive function or short-term memory^38^. One plausible explanation is the heterogeneity of included patient populations and especially the heterogeneity of applied cognitive interventions^2,34,38^.

Indeed, the efficacy of cognitive training interventions critically depends on design choices such as the type of intervention (e.g., computerized cognitive training; content of training), delivery format (e.g., group or home-based training), dosing (session length and frequency, total duration) and training adherence^26^. The present study used a training duration of 14 weeks, with 3 trainings per week and 40 trainings overall, with one training session lasting 30 minutes. Training was home-based, but training adherence was supervised, and assistance was provided via e-mail or telephone if needed. Additionally, participants were able to communicate among each other and with the study team via a web-based community platform. In comparison to previous studies, the training period of the present study was longer, more intensive (except Kesler et al^31^., who had similar intervals of training sessions per week) and with the largest variety of tasks and gamification. The duration of one individual training session was in the lower range compared to other studies^27,29,31,32,39^.

The most efficient design is currently a subject of intensive research and some conflicting evidence exists. However, 3 trainings per week with 30 minutes session length and 20 hours total training duration—as applied in the current study—have been shown to be highly effective^26,40^. In addition, training adherence was excellent in our study for the full course of the program and drop-out rate was low, most likely due to diversified training sessions and the gamification approach. Indeed, a recent meta-analysis showed that gamification in CCT is more demanding for participants then CCT without gamification, but comes with the important benefits of higher motivation and engagement that in turn increases adherence and training efficacy^41,42^. Training in our study was easy to attend from the patients‘ own computer, and weekly email reminders by our study team were provided, including an additional reminder if a training session was missed. As reported by the short 4-items questionnaire, participants’ satisfaction with the training was high and almost all patients would have participated again (Supplementary Table 2).

Importantly, in comparison to previous studies, the current study investigated cognitive impairment in breast cancer patients during the immediate post-treatment phase with a mean of 6.6 (SD 9.3) months after adjuvant treatment, a vulnerable phase, in which up to 75% of patients report CRCI. Indeed, recent evidence suggests that cognitive treatment interventions should ideally target these disease stages given that the reduction in quality of life and perceived cognitive abilities is most severe during the early period after primary breast cancer treatment and improves over years^2,4,34,35^. However, most previous studies assessed the effects of CCT in breast cancer at later stages, i.e., after a mean of 46 up to 72 months following therapy^27,29,31,33,39^. Our results show that cognitive training in breast cancer patients is both feasible and may be effective already 7 months after primary treatment.

More generally, an increasing number of studies has now shown effectiveness of CCT on cognitive function in a wide range of diseases, including CRCI in patients with many different types of cancer^26,34,43^. CCT mainly improves processing speed, executive function, working memory, cognitive flexibility, language and immediate and delayed memory^24–26,44^, but can also improve perceived cognitive impairment and quality of life^45^. In a recent meta-analysis, 11/16 analyzed studies reported an improvement in objective cognitive function, and 8/16 reported improvement of subjective cognitive complaints after CCT in cancer survivors with CRCI^44^.

In contrast to improved cognitive function in objective assessments, patients did not improve in subjective cognitive performance or quality of life in our study. Indeed, recent evidence shows only limited correlation between objective and subjective cognitive impairments. In line with our results, some studies report improvement of objective cognitive deficits without improvement of subjective cognitive function following cognitive training^46^, while other studies observed the opposite effect^45^, and yet other studies report improvement of both subjective and objective cognitive impairment after training^27,31^. Reasons for these varying results may include the different perception of impairment in different patient populations and a differential role of factors impacting quality of life in addition to cognitive dysfunction, e.g., ongoing worry about the disease, fatigue and pain. Since our study was conducted during the immediate post-treatment phase, these factors may be more present than in studies performed later after initial treatment.

While the majority of current studies focus on breast cancer survivors, recent evidence indicates that CCT is effective in other tumor entities and patient populations, including pediatric cancer, brain tumors and prostate cancer^44,47–52^. Larger clinical trials are now needed to define intervention timepoints and ideal training designs for clinical routine application of cognitive training in these different patient populations.

The current study assessed CCT in the immediate post-treatment phase. However, this early phase after primary cancer treatment may be characterized by heightened emotional distress and physical fatigue^53,54^, which could impede engagement and adherence to cognitive interventions. Although in the present study adherence was excellent, this should be considered for further investigation or when implementing CCT in clinical settings. Additionally, there may be concerns regarding the potential interference of cognitive interventions with concurrent cancer treatments or the exacerbation of treatment-related side effects. Therefore, careful consideration of these factors is warranted when implementing cognitive interventions in the immediate post-treatment phase, and close monitoring of patients’ physical, emotional, and cognitive well-being is essential to ensure the intervention’s safety and efficacy.

This trial compared CCT to a passive control group. This design choice was guided by synthesis of a large body of evidence robustly showing a limited moderating role for the type of control condition (i.e., sham vs passive control) in randomized controlled trials of cognitive training^26,55,56^. However, this design choice does not allow us to indicate whether CCT effects will be superior to those of other potentially efficacious interventions or to non-specific cognitive activities.

At baseline, significantly more participants in the control group than in the training group received hormone therapy (100 vs. 75%); at follow-up, group differences were not significant. While evidence indicates that hormone therapy can impact cognitive function and can be associated with fatigue and mood changes^14,15,57^, we observed no group difference between the training and control group at baseline regarding cognitive performance, fatigue levels and depressive symptoms in our study.

Strengths of our study include the training design and dosing that was based on recent evidence for achieving high training efficacy, the excellent training adherence, the age- and education-matched control group and the similar distribution of tumor characteristics and treatments and the training and control group. Limitations are the small sample size and the heterogeneity of participants regarding time since therapy. Interestingly, we observed no impairment of verbal memory at baseline which might be related to the applied task or the small sample size. However, we were nevertheless able to observe significant improvements of cognitive function following cognitive training in breast cancer patients.

Web-based cognitive training may improve overall cognitive impairment and executive function in breast cancer patients. Our results shows that computerized cognitive training is effective in the immediate post-treatment phase. These results should be verified in larger clinical trials with carefully selected training dosing strategies and measures to ensure high level of training adherence.

## Methods

We recruited 40 breast cancer patients from seven breast cancer centers in the Berlin-Brandenburg area. Inclusion criteria were: (i) histologically confirmed primary breast cancer; (ii) age 18–80 years; (iii) time since primary breast cancer treatment (surgery and additional treatments including chemotherapy, radiation or antibody therapy) of up to 30 months; (iv) presence of subjective cognitive impairment in the form of attention and memory disorders. Exclusion criteria were: (i) male sex; (ii) recurrent or metastatic cancer; (iii) current relevant neurological or psychiatric disorder (including clinical depression; patients with currently clinically irrelevant disorders were not excluded). In line with other recent studies, current hormone therapy was not an exclusion criterion^29^. According to these inclusion and exclusion criteria, 34 patients were eligible to participate in the study. The study was conducted in accordance with the ethical standards of both the institutional and national research committees, including the Declaration of Helsinki^58^. The study was approved by the local ethics committee “Ethikkommission der Charité—Universitätsmedizin Berlin” and all patients gave written informed consent.

Participants were randomized into a training group (*n* = 17) or a control group (*n* = 17) on a 1:1 basis with stratification for age and years of education. After the start of the web-based training, 3 patients dropped out (control group: lack of time (1), recurrent cancer (1); training group: technical issues (1)). The remaining 31 patients (control group, *n* = 15; training group, *n* = 16) completed all training sessions.

**Fig. 4:**
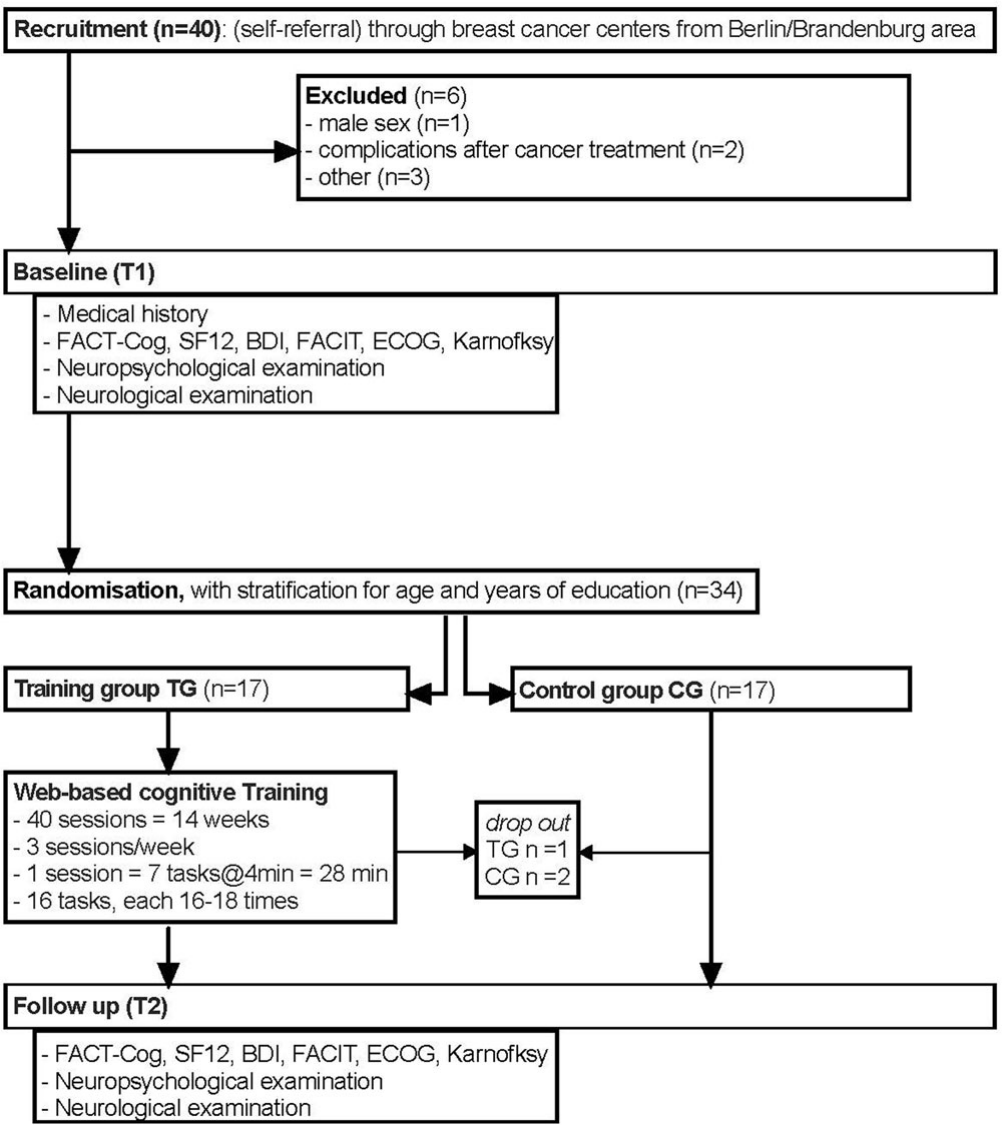
Study design. Overview of the study protocol including recruitment, randomization, intervention period and data acquisition at baseline (T1) and follow up (T2).

Study visits were performed at study initiation (Baseline, Timepoint 1, T1) and follow-up after the training (5 months after baseline visit; Timepoint 2, T2) and included neuropsychological assessment, assessment of subjective cognitive impairment and neurologic examination (Fig. 4).

### Neuropsychological assessment

Neuropsychological assessment was performed with a battery of cognitive tests covering verbal learning and word fluency, memory, intelligence level, attention and executive function. At follow-up, the appropriate alternate versions of the tests were used. Six cognitive domains were tested: Verbal learning was examined using the Verbal Learning Memory Test (VLMT), the German equivalent to the Rey auditory verbal learning test. The Rey-Osterrieth Complex Figure was used to assess visuospatial memory. Short-term memory and working memory were evaluated with the digit span forwards and backwards trials from the Wechsler Memory Scale. The subtest 3 of the Leistungspruefsystem (LPS), the German equivalent to Raven’s Progressive Matrices, was used to evaluate fluid intelligence and the Mehrfachwahl-Wortschatz-Intelligenztest-A (MWT-A), the German equivalent to the National adult reading test (NART), was used to test crystallized and premorbid intelligence. For assessment of attention, the tonic and phasic alertness tasks, and the divided attention task from the computerized test battery for attention assessment (TAP) were applied. Executive function was studied with the TAP Go/NoGo task and the Stroop test. Word fluency was evaluated using the Regensburger word fluency test.

Based on the criteria of the International Cancer and Cognition Task Force^59^, cognitive impairment for single cognitive tests, cognitive domains (verbal learning, visuospatial memory, working memory, fluid intelligence, attention, executive function) and overall cognitive impairment was defined as test performance of −1.5 standard deviations in at least two cognitive tests or −2 standard deviations in at least one test. For single cognitive tests, normative control of the respective test system, using age and education adjusted standard values obtained from raw data, were used to identify test deficits.

### Subjective cognitive impairment

Subjective cognitive impairment was examined using the Functional Assessment of Cancer Therapy-Cognitive Function (FACT-Cog) Version 3 with the four subscales perceived cognitive impairment (PCI), perceived cognitive ability (PCA), quality of life (QOL) and comments from others (OTH); PCI was used as recommended as a primary score^60^. A composite score of the subscales, the FACT-Cog cognitive function score, was also calculated^61^. In addition, subjective cognitive deficits were assessed with a short semi-structured interview focused on general cognitive impairment in daily life. Items were similar to items from the European Organization for Research and Treatment of Core Quality of life Questionnaire (EORTC-QLC-C30, Item Number 20 and Item Number 25). Patient-reported deficits were classified into the domains memory, learning, and attention, or a combination of these.

### Patient-reported outcomes (PROMs)

Questionnaires for assessment of quality of life, health status, fatigue and depression included the 12-Item Short Form Health Survey (SF-12), the Eastern Cooperative Oncology Group scale of performance status (ECOG), the Karnofsky performance status, the QOL subscale of FACT-Cog Version 3, the Functional Assessment of Chronic Illness Therapy—Fatigue (FACIT-Fatigue), and the Beck Depression Inventory—Fast Screen (BDI-FS).

### Neurologic examination

A comprehensive neurological status including assessment of the cranial nerves, motor function, sensitivity and assessment of the ability to stand, walk and coordinate was examined.

### Web-based cognitive training intervention

The training group underwent a cognitive training for 14 weeks. The web-based training was provided by NeuroNation (Berlin, Germany) and adapted for the purpose of this study. Training was home-based and performed on participants’ private computers. Only one training per day was allowed. Training adherence was supervised online, and telephone and e-mail-based support as well as training reminders were provided by our study team. Additionally, participants were able to use a web-based community platform with the study team to provide help with technical or content-related problems as well as to strengthen the participants’ sense of community and thus increase adherence.

The training included 16 tasks, addressing the cognitive domains attention, working memory and executive function—based on the domains most affected in breast cancer patients in previous studies^3,5,6^. The whole concept of the cognitive training was based on gamification, tasks were presented like a computer game, task difficulty was dynamically adapted based on participants’ performance to provide a continuous training challenge and to enhance training motivation (Supplementary Table 3). Each training session included 7 of the 16 tasks. One task lasted 4 minutes and one session ~30 minutes. Patients trained three sessions per week and were free to choose the training days during the week, training on consecutive days was allowed. The full training consisted of 40 sessions per patient for a period of 3 months with every task occurring 16–18 times during this training period.

To encourage study participation at T2, all patients in the control group were given access to the training program after completion of neuropsychological testing at T2 and the end of all data acquisition. At timepoint 2, participants of the training group answered a 4-items training evaluation questionnaire (adapted from Schmiedek et al., 2010), with the following questions: *“Do you think that a web-based cognitive training program could improve cognitive deficits in breast cancer patients?”, „Did you have fun participating in the study?“, „Would you recommend participating in the study to others?“, “Would you do it again?”*^36^.

### Statistical analysis

Age and years of education were compared with Mann-Whitney-U-Test. Demographic factors and treatment forms were analyzed with Pearson’s chi-square test. For FACT-Cog, a cognitive function score was computed by summarizing all four subscale scores of Fact Cog (PCI, PCA, OTH and QOL); subscales were analyzed individually and primary score was PCI^60^.

Analysis of longitudinal change for FACT-Cog items from T1 to T2 was performed by ANCOVA, with the individual item at T2 as the dependent variable, with baseline adjustment for its T1 scores and age as covariates. FACT-Cog subscale scores, FACT-Cog cognitive function score and measures of quality of life (SF-12, FACIT, BDI, ECOG, Karnofski, FACT-Cog-QOL) were compared between groups at T1 and T2 with t-tests. Comparison of cognitive impairment rates between groups at T1 and again at T2 was performed with Pearson’s chi-square test for cognitive impairment and cognitive domain deficits. Improvement or decline of cognitive impairment rates overall and for cognitive domains from T1 to T2 was analyzed using McNemar’s test. For Pearson’s chi-square test and McNemar’s test Phi coefficient was used as measure of effect size with *φ* = 0.1 small effect, *φ* = 0.3 intermediate effect and *φ* = 0.5 strong effect as cutoff values^62,63^. Differences between groups were interpreted as significant if *p* < 0.05. Statistical analysis was performed with IBM SPSS Statistics 25.

## Supplementary information

Supplementary Material

## Data Availability

The data that support the findings of this study are not openly available due to reasons of data protection and are available from the corresponding author upon reasonable request. Data are located in controlled access data storage at Charité Universitätsmedizin Berlin.

## Code availability

The code used for statistical calculations in this analysis is available from the corresponding author upon reasonable request.
