# Supplementary material for: Computerized cognitive training improves cognitive function in primary breast cancer survivors
Source: NPJ Breast Cancer. 2024 Sep 30;10:85. doi: 10.1038/s41523-024-00694-8 (PMC11443049; doi:10.1038/s41523-024-00694-8)
Supplement: Supplementary file 1 — Supplementary Material [file 41523_2024_694_MOESM1_ESM.pdf]

**Supplementary Table 1: Baseline history of disorder**					
	**Control group (n=15)**		**Training group (n=16)**		
	**N**	**%**	**N**	**%**	**p-value**
**Neurological**	4	27	7	44	0.32
**Psychiatric**	2	13	3	19	0.68
**Rheumatological**	0	0	1	6	0.33
**Autoimmune**	0	0	1	6	0.33
**Haematological**	0	0	1	6	0.33
**Dermatological**	0	0	2	13	0.16
**Cardiac**	1	7	1	6	0.96
**Pulmonary**	2	13	3	19	0.68
**Gastroenterological**	0	0	2	13	0.16
**Gynaecological**	3	20	4	25	0.74
**ophtalmological**	0	0	0	0	-
**Urological**	1	7	1	6	0.96
**ETN**	1	7	0	0	0.29
**Thyroid**	2	13	3	19	0.68
**Endocrinological**	3	20	4	25	0.74
**Family history of cancer**	10	67	13	81	0.27

Supplementary Table 1: Baseline history of disorder. Depicts history of disorder and family history of cancer at baseline. Percentages are rounded.

**Supplementary Table 2: Training evaluation (n=16)**				
	“Do you think that a web-based cognitive training program could improve cognitive deficits in breast cancer patients?”	*„Did you have fun participating in the study?“*	*„Would you recommend participating in the study to others?“*	*“Would you do it again?”*
Yes	14	13	14	15
No	1	3	0	1
No comment	1	0	2	0

Supplementary Table 2: Training evaluation of participants of the training group at follow up (T2). Adapted from Schmiedek et al., 2010.

Supplementary Table 3: Neuronation training tasks			
**Task name**	**Task description**	**Targeted cognitive domain**	**Screenshot**
**Colorado**	Click on the card that is being searched for according to the task “colour” or “meaning”. Sometimes it is also displayed what is NOT searched for	Working memory, sustained attention	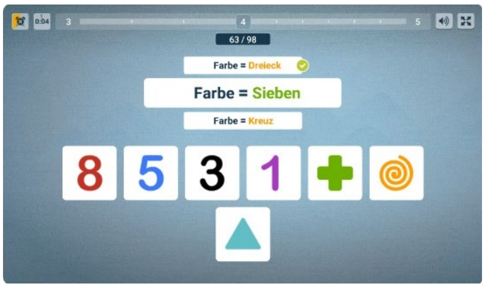
**Signpost**	Use the arrow keys on your keyboard to start the exercise and move the ball. Follow the arrows on the way up. The middle arrow is the directional one. If one arrow is displayed brighter than the others, it is the directional one. Collecting the stars will give you more points	Sustained attention	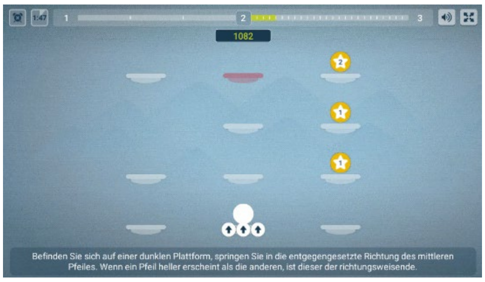
**Rhythm master**	Points appear one after the other on the turntables. The new points are coloured white for one full rotation. With white points, entry using the arrow key is not necessary. As soon as a white dot crosses the active area for the second time and turns blue, the corresponding arrow key on your keyboard	Working memory, sustained attention	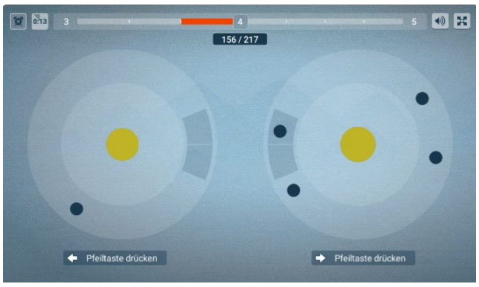

	must be pressed and, if done correctly, a rhythm will be created		
**Mackworth-Clock**	Observe the clockwise points that the pointer points to light up one after the other. Whenever a pointer skips a point, you must react. To do this, click on "Jump" ("Sprung"). Your full concentration is required when you have to pay attention to more than one hand	Working memory, spatial attention	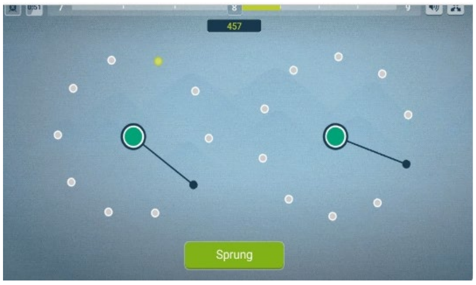
**Chain reaction**	Click alternately on the elements shown above. If you should lose the thread, you can orientate yourself on the elements clicked on last. Observe the logical rules of the chains	Working memory, spatial and sustained attention	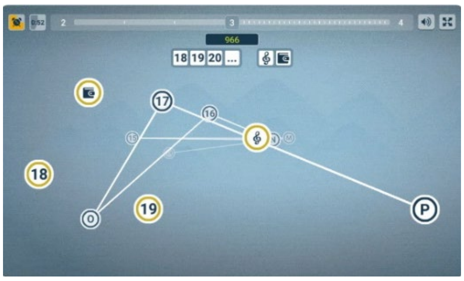
**Formfever-Express**	You see two rows of boxes with questions. Evaluate the shapes that appear with respect to the particular question and click "Yes, true" or "No"	Working memory, sustained attention	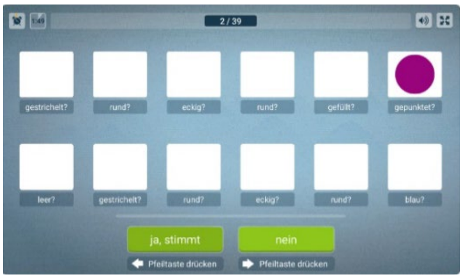

**Colour confusion**	You see a word written in colour at the top. Pay attention only to the colour of the writing, not to the meaning of the word. Click the word in the selection that matches the color of the top word. Ignore the color of the lower word.	Sustained attention	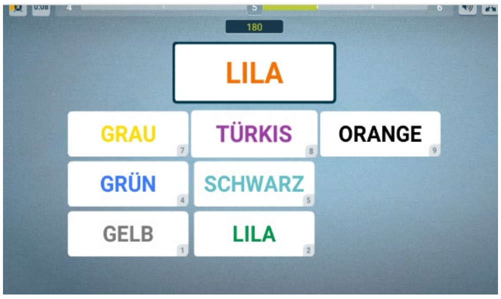
**Flash**	Click the numbers in ascending order. React quickly. Gradually, more numbers appear and the field becomes more confusing	spatial and sustained attention	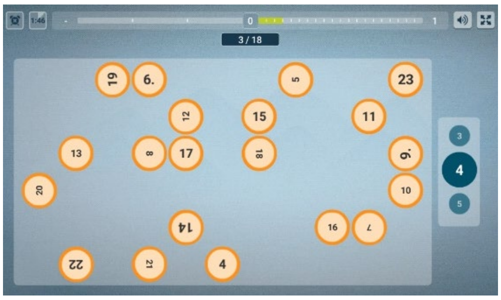
**Eagle eye**	Find the largest number among the moving digits. You can enter it using the number field on the right or click on it directly	Sustained attention	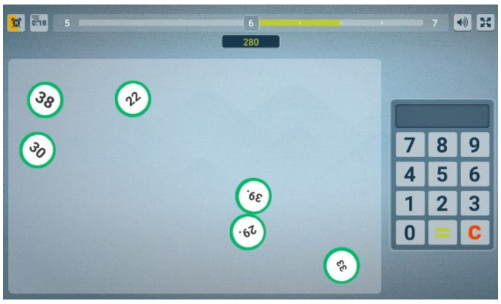
**Multiple memory**	Several colored balls land in the boxes and leave them again. Note how many balls of each color remain in the boxes. Indicate the number of balls in each box in turn	Executive function, sustained attention	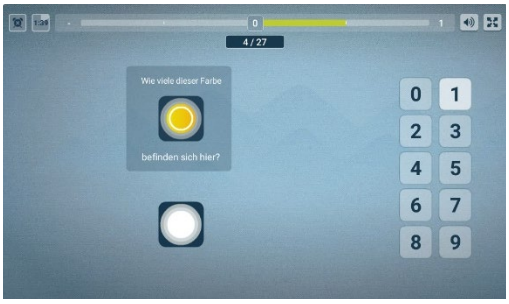

**Mirror image**	Memorize the position of the blue box on the grid. Click on the "Remembered" field to continue. Now decide whether the shape in the grid is vertically symmetrical, asymmetrical or horizontally symmetrical. Here it is asymmetrical. Remember the position of another blue box on the grid. Do not forget the first position! At the end of the round, click the memorized positions in the grid	Executive function, working memory, concentration	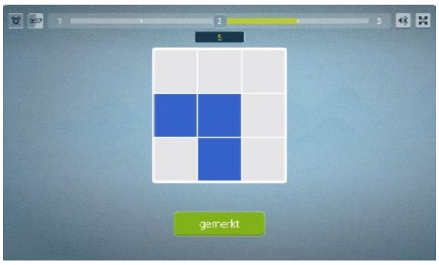<br>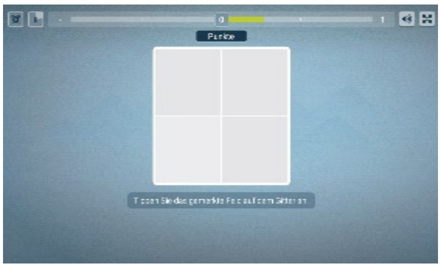<br>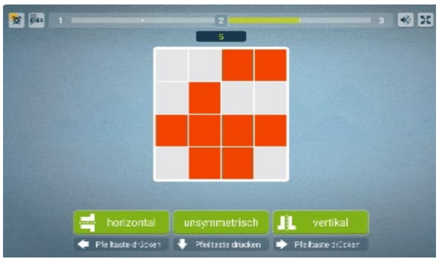
**Backfinder**	At the beginning of each round you will be shown a path. Please remember which points are connected by this path one after the other. The path disappears again and the last connected point is marked with a circle. Connect the points one by one in the reverse order as you saw them at the beginning. Start with the point marked by a circle	Working memory sustained attention	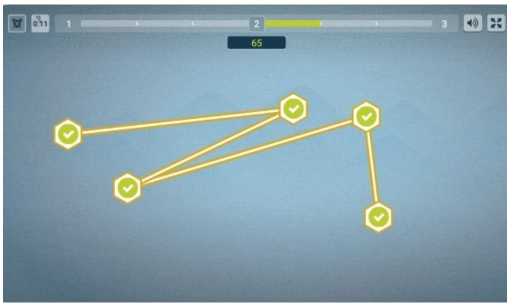

**Serial memory**	Remember the number or letter that appears. Now solve the given task in your head and click on "Solved". Specify whether the displayed number is less than, equal to, or greater than the result of the calculation task. Finally enter the two memorized numbers or letters.	Working memory	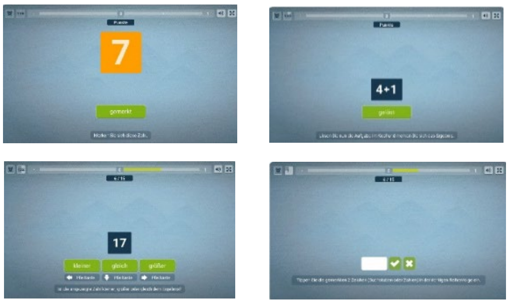
**Parallel calculator**	Calculate the displayed task and enter the result. Remember the result well! Now apply the given number to the last calculated result of each box. The box now disappears and you have to remember the result. A second box appears with a new task that you have to solve. Now remember this result well! Next, the first box will appear again and you have to continue calculating with the result that you should remember first. Then the second box will appear again and you continue calculating there	Working memory	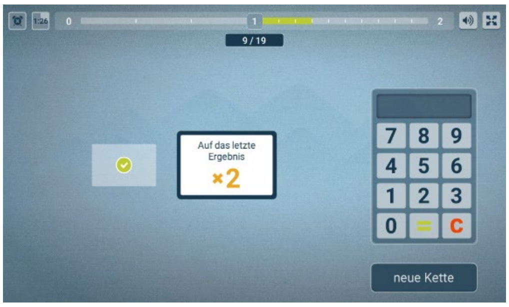

	with the second result		
**Parita**	For this exercise it is necessary to turn on the sound of your computer. Memorize the number shown until the end of the exercise. You only need to keep the symbol shown in your mind for a short time. Click on the "Remembered" field to continue. The symbol disappears and a second symbol appears next to it. Decide whether the symbols are identical. If the symbols are identical or the number heard matches the one shown at the beginning, click the "Sound or Image" field. If neither the symbols are identical, nor the number heard matches the one shown at the beginning, you don't need to do anything	Working memory	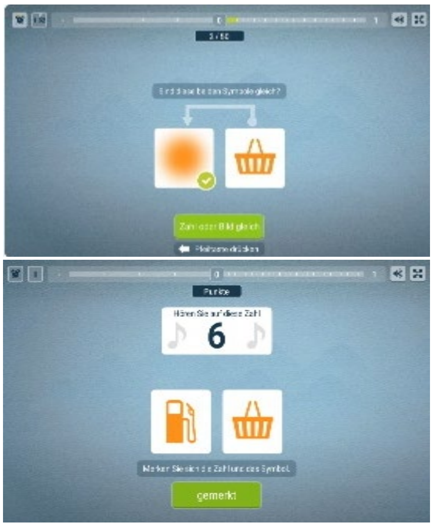

Supplementary Table 3: Neuronation training tasks. Depicts name and short description of each task, the targeted cognitive domain and a screenshot of each task.
